# Searching in Mother Nature for Anti-Cancer Activity: Anti-Proliferative and Pro-Apoptotic Effect Elicited by Green Barley on Leukemia/Lymphoma Cells

**DOI:** 10.1371/journal.pone.0073508

**Published:** 2013-09-09

**Authors:** Elisa Robles-Escajeda, Dennise Lerma, Alice M. Nyakeriga, Jeremy A. Ross, Robert A. Kirken, Renato J. Aguilera, Armando Varela-Ramirez

**Affiliations:** 1 Department of Biological Sciences and Border Biomedical Research Center, the University of Texas at El Paso, El Paso, Texas, United States of America; 2 St. Mary’s University School of Science, Engineering and Technology, San Antonio, Texas, United States of America; 3 Department of Biomedical Sciences, Texas Tech University Health Sciences Center, El Paso, Texas, United States of America; Wayne State University School of Medicine, United States of America

## Abstract

Green barley extract (GB) was investigated for possible anti-cancer activity by examining its anti-proliferative and pro-apoptotic properties on human leukemia/lymphoma cell lines. Our results indicate that GB exhibits selective anti-proliferative activity on a panel of leukemia/lymphoma cells in comparison to non-cancerous cells. Specifically, GB disrupted the cell-cycle progression within BJAB cells, as manifested by G2/M phase arrest and DNA fragmentation, and induced apoptosis, as evidenced by phosphatidylserine (PS) translocation to the outer cytoplasmic membrane in two B-lineage leukemia/lymphoma cell lines. The pro-apoptotic effect of GB was found to be independent of mitochondrial depolarization, thus implicating extrinsic cell death pathways to exert its cytotoxicity. Indeed, GB elicited an increase of TNF-α production, caspase-8 and caspase-3 activation, and PARP-1 cleavage within pre-B acute lymphoblastic leukemia Nalm-6 cells. Moreover, caspase-8 and caspase-3 activation and PARP-1 cleavage were strongly inhibited/blocked by the addition of the specific caspase inhibitors Z-VAD-FMK and Ac-DEVD-CHO. Furthermore, intracellular signaling analyses determined that GB treatment enhanced constitutive activation of Lck and Src tyrosine kinases in Nalm-6 cells. Taken together, these findings indicate that GB induced preferential anti-proliferative and pro-apoptotic signals within B-lineage leukemia/lymphoma cells, as determined by the following biochemical hallmarks of apoptosis: PS externalization, enhanced release of TNF-α, caspase-8 and caspase-3 activation, PARP-1 cleavage and DNA fragmentation Our observations reveal that GB has potential as an anti-leukemia/lymphoma agent alone or in combination with standard cancer therapies and thus warrants further evaluation *in vivo* to support these findings.

## Introduction

Globally, barley is considered a non-toxic plant [1] that produces a cereal grain that serves as a base malt in the brewing industry. It is also a healthy component of various foods and beverages (bread, soups, stews, beer, etc.) and as major animal forage. Independent of its grain, 10- to 12-inch-long young barley leaves, also referred to as green barley, are ingested as an infusion and are also prepared for human consumption as dried powder. Young barley leaves are recommended as a dietary supplement because of their vitamin and mineral content [2].

Previous studies have indicated that extracts from whole barley kernels exhibit anti-oxidant and anti-proliferative effects on human colorectal cancer Caco-2 cells [3]. Nevertheless, the anti-proliferative activity within green barley leaves remains to be elucidated. Green barley products have anti-inflammatory properties and can modulate tumor necrosis factor-alpha (TNF-α) production/release on human monocyte THP-1 cells [4]. Similarly, another study reported that a compound isolated from green barley leaves possessed anti-oxidant properties [5]. Furthermore, small molecules (less than 1 kDa) purified from green barley extract (GB) inhibited TNF-α release from mononuclear cells obtained from rheumatoid arthritis (RA) patients, suggesting that GB could be a natural drug with anti-oxidant and anti-inflammatory activity that alleviates the symptoms of patients afflicted with RA [6]. Purification studies were conducted using advanced methods to characterize the specific compounds that are responsible for the observed biological activities of GB. Markham and Mitchell showed that the flavone-c-glycosides, saponarin and lutonarin, from young green barley leaves were responsible for the anti-oxidant properties [7]. Similarly, biomasses from green barley plants possess significant quantities of the anti-oxidant enzymes catalase and superoxide dismutase, as well as the non-enzymatic anti-oxidants vitamins C and E [8,9]. Consistent with these observations, *in vivo* studies involving 36 subjects suggested that daily supplements of barley leaves in combination with anti-oxidant vitamins (C and E) decreased the low-density lipoprotein (LDL)-vitamin E content and inhibited small dense-LDL oxidation, consequently reducing some of the major risk factors of atherosclerosis and protecting type 2 diabetic patients against vascular diseases [10]. Furthermore, a combination of saponarin/lutonarin (4.5/1 proportion) isolated from young barley leaves was found to have anti-oxidant effects that were comparable to those obtained from α-tocopherol and butylated hydroxytoluene [11].

It has been proposed that the anti-oxidant and anti-cancer activities in fruit and vegetables are attributable to the additive or synergistic consequence of their complex mixture of phytochemical components [12]. Moreover, the total polyphenol fraction within cranberries exhibited more efficient anti-proliferative activity compared with its individual components, suggesting a combined additive or synergistic influence [13]. In addition, several studies have revealed that plant products can act as cell cycle suppressing agents, interrupting the initiation or progression phases of carcinogenesis [14–17]. Furthermore, it has been noted that cancer patients often ingest plant products in addition to their prescribed medicines [18] based on an assumption that the plant products have innocuous side-effects and are a well-studied therapeutic choice.

Despite evidence of GB’s potential as an anti-inflammatory mediator, there is meager evidence of its direct anti-proliferative and/or cytotoxic activity on normal or transformed cells. In this study, we sought to examine the anti-proliferative and cytotoxic activity of GB on various leukemia/lymphoma cell lines. Our data demonstrate that GB has selective anti-proliferative effect on several leukemia/lymphoma cells, with little if any on non-cancerous cells. Of four cancer cell lines, pre-B (Nalm-6) and mature-B (BJAB) cells were the most sensitive to GB’s anti-proliferative activity. For the first time, our study showed that GB led to apoptotic-induced cell death through TNF-α release, caspase-8 and caspase-3 activities, PARP-1 cleavage, PS translocation, cell cycle arrest-associated DNA fragmentation. These studies provide support for the potential utility of GB against leukemia/lymphoma and warrant further investigation in animal model systems.

## Materials and Methods

### Green barley extracts (GB) preparation

Green barley powder from young leaves of *Hordeum vulgare* L., sold commercially as an herbal supplement as a proactive source of vitamins and minerals (Vitamin World; www.vitaminworld.com), was utilized. Dehydrated GB powder was resuspended with phosphate buffered saline (PBS; Life Technologies, Grand Island, NY) at a concentration of 10% w/v. The rehydrated powder suspensions were then exposed to three consecutive freeze/thaw cycles at -80 °C/room temperature, respectively. Then, the samples were repeatedly sonicated (Vibra Cell; Sonics and Materials Inc., Newtown, CT) at maximum amplitude 40%, ~ 14 watts, using a ¼” microtip, by a 20 sec sonication pulse followed by 30-sec rest intervals, for seven successive cycles. Tubes containing the suspension mixtures were kept pre-chilled in an ice-water bath throughout the procedure to prevent heating. After centrifugation at 15,000×*g* for 30 min, the supernatants were collected and aseptically filtered, initially through 0.45-µm membrane pores, followed by a second filtration through 0.22-µm membrane pores (Cole-Parmer, Chicago, IL). Samples of sterile PBS without GB were utilized as controls. Additionally, the dry weight of GB soluble material was measured to determine the concentration of plant dry weight, in mg/ml, used in each treatment. Three aliquots of 1 ml from both GB samples prepared in PBS were lyophilized (Labconco 4.5 L freeze dryer, Stanford, CA) overnight. Additionally, aliquots of 1 ml PBS alone were used to subtract the PBS solvent contribution, and the dry weight was calculated. Typical volumes used in this study and their dry weight equivalents of GB are depicted in Table S1.

### Cell lines and culture conditions

Four human leukemia/lymphoma cell lines were utilized: YT Natural Killer-like [19], mature-T acute lymphoblastic leukemia Jurkat [20], pre-B acute lymphoblastic leukemia Nalm-6 [21], and mature-B Burkitt’s lymphoma BJAB [22]. For comparative purposes, one cell line from non-cancer origin, human dermal neonatal foreskin Hs27 fibroblasts (Hs27; ATCC, Manassas, VA), was also included. The culture media for leukemia/lymphoma (YT, Jurkat, Nalm-6 and BJAB) and fibroblast (Hs27) cells were RPMI and DMEM (HyClone, Logan UT), respectively. Both of these culture media were supplemented with 10% heat-inactivated fetal bovine serum (Hyclone), 100 U/ml penicillin, 100 µg/ml streptomycin, and 0.25 µg/ml amphotericin B (Lonza, Walkersville, MD). Exponentially growing cells, at approximately 60-75% confluence, were counted and seeded into 24-well plates. The incubation conditions of the cells were 37^°^C in a humidified 5% CO_2_ atmosphere. To ensure high viability, cells were processed as previously described [23].

### Anti-proliferative assay

Leukemia/lymphoma and non-cancer cells were seeded into a 24-well plate format at 1x10^5^ cells/well and 1.25x10^4^ cells/well in 1 ml media, respectively. After the cells were exposed to 50 µl/ml of GB for 96 h, the absolute cell number per milliliter was quantified using an inverted microscope and a Neubauer chamber (hemocytometer), to estimate anti-proliferative activity compared with untreated negative controls. Cells growing in suspension were directly homogenized, and an aliquot was loaded onto a hemocytometer and counted. For adherent Hs27 cells, the supernatant containing the floating cells (mainly dead) from each well were collected in a tube, whereas the adherent cells were detached by trypsinization as previously detailed [24]. Floating and adherent cells were homogenized and counted, as detailed above. The results are expressed as the average of quadruplicate cultures. The PBS-treated controls, diluent of the GB, were normalized to 100% and used as a reference to calculate the percentage of GB-treated cell proliferation.

### Cytotoxicity monitored by vital dye propidium iodide exclusion assay

For the quantification of cytotoxicity, cell samples were collected, stained with the membrane-impermeant dye propidium iodide (PI) and monitored in live-cell mode *via* flow cytometry (Cytomics FC500; Beckman Coulter, Miami, FL). This assay identifies single PI-positive cells with disrupted plasma membranes that are considered dead cells. Approximately 10,000 events were collected for each sample, and the data were analyzed as previously reported [25]. As positive anti-proliferative/cytotoxic controls, cells were exposed to 1 mg/ml G418, a protein synthesis inhibitor. Additionally, as negative controls, cells treated with equivalent volumes of GB diluent alone, PBS, and untreated cells were included in parallel cultures. The data acquisition and analysis was performed using CXP software (Beckman Coulter).

### Cell cycle analysis by measuring cellular DNA content

Analysis of the cellular DNA content and cell-cycle distribution was performed by PI staining and monitoring its fluorescence *via* flow cytometry. The influence of GB as a possible cell-cycle disruptor was investigated on asynchronous BJAB cell line after 96 h of incubation. Cells were seeded on a 24-well plate, cultured as described above and harvested as previously detailed [24]. Cell nuclei were prepared using the DNA-Prep Coulter reagents kit (Beckman Coulter) following the manufacturer’s instructions. Briefly, cells were harvested and centrifuged at 262xg for 5 min. The supernatant was discarded and then the cell pellets were gently resuspended by low-speed vortex using 100 µl DNA-Prep lysis and a permeabilization reagent (detergent), followed by the addition of 400 µl DNA Prep stain reagent (50 µg/mL PI and 4 kU/mL RNAse). Subsequently, the samples were incubated at room temperature in the dark for 60 min, followed by flow cytometry analysis (LSRII; BD Biosciences, San Jose, CA). As a positive control for cell-cycle arrest, two independent experiments were performed in which cells were exposed to 1 mg/ml G418 and 80 nM etoposide (Sigma-Aldrich, St Louis, MO) for 96 h. As a control for non-specific effects, the diluent of GB, PBS (10 and 50 µl), as contained in the experimental samples, was included. Untreated cells were used as controls. The percentages of cells with different DNA content distributions were determined from histogram with gates for sub-G0/G1, hypodiploid; G0/G1, diploid; S, hyperdiploid; and G2/M, tetraploid. Gating was applied to exclude doublets. The S-phase population was defined as the percentage of cells with a DNA content between G0/G1 (diploid) and G2/M (tetraploid). Additionally, this type of analysis was used for its ability to detect an apoptosis-associated DNA fragmentation pattern at the single cell level, as manifested by an increase in the sub-G0/G1 cell subpopulation [26]. To deconvolute the DNA content histograms, FACS Diva (BD Biosciences) or FlowJo (Tree Star, Ashland, OR) software was utilized.

### Analysis of phosphatidylserine (PS) distribution in cellular membranes

To investigate whether disruption of cellular membrane PS asymmetry is involved as a mechanism of cell death induced by GB, cells were monitored *via* flow cytometry after dual staining with annexin V-FITC and PI. Cells were seeded in 24-well plates t at a density of 100,000 cells per well in 1 ml of culture media. After overnight incubation, the cells were treated with 50 µl of GB or PBS control for an additional 48 h and 96 h, for Nalm-6 and BJAB cells, respectively. The cells were harvested, washed with cold PBS and processed following the manufacturer’s instructions (Beckman Coulter). Briefly, cells were stained with a solution containing a mixture of annexin V-FITC and PI in 100 µl of binding buffer, incubated on ice in the dark for 15 min, followed by addition of 400 µl of ice-cold binding buffer and immediately analyzed *via* flow cytometry (Cytomics FC 500; Beckman Coulter). The total percentage of apoptotic cells was defined as the sum of both early and late phases of apoptosis (annexin V-FITC positive), top and bottom right quadrants in a flow cytometric dot plots, respectively. For each sample, 10,000 events were collected and analyzed utilizing CXP software (Beckman Coulter). Each experimental point and controls were assessed in quadruplicates.

### Live-cell detection of intracellular caspase-3 activation

Cysteine-aspartic proteases (caspase)-3 activation in Nalm-6 GB-treated cells was measured using a fluorogenic NucView 488 Caspase-3 kit for live cells (Biotium, Hayward, CA) according to the manufacturer’s instructions. The cells displaying a green fluorescence signal were monitored *via* flow cytometry (Cytomics FC500). The cells were seeded on a 24-well plate format as described above and exposed for 6 h and 8 h to 50 µl GB extract. Several controls were included in this series of experiments: cells treated for 8 h with GB extract were exposed to 10 µM Ac-DEVD-CHO (DEVD; N-Acetyl-Asp–Glu–Val–Asp-aldehyde), a potent, specific and irreversible inhibitor of caspase-3 [27], before the addition of NucView substrate; cells treated for 8 h with 2 µg/ml camptothecin, a well-known inducer of apoptosis *via* caspase-3 activation [26], as a positive control; and untreated cells were used as a negative control. Data collection and analysis of the caspase-3-positive cells was performed using CXP software.

### Western blot analysis of PARP-1 cleavage

The cleavage of poly(ADP-ribose) polymerase-1 (PARP-1) was assessed by Western blotting as detailed previously [28]. Briefly, 50 µl or 100 µl GB was added to 1x10^5^ Nalm-6 cells in 1 ml media and incubated for 24 h. As a positive control of PARP-1 cleavage induction, 2 µg/ml camptothecin was used. A potent inhibitor of caspase-3, Ac-DEVD-CHO (20 µM), was added to the cells concurrently with GB to determine the contribution of caspase-3 in PARP-1 cleavage. Equal amounts of the cell extracts, 100 µg protein per lane in Laemmli-reducing buffer, were separated using a 10% mini-gel (Bio-Rad, Hercules, CA) for SDS-PAGE. Protein concentrations of the cell extracts were quantified with the bicinchoninic acid protein assay kit (Pierce, Rockford, IL). After electrophoresis, proteins were transferred from the gel onto a polyvinylidene fluoride (PVDF) membrane (Thermo Fisher Scientific Inc., Rockford, IL) as described previously [29]. Next, PVDF membrane blots were blocked with 5% (w/v) skim milk powder in TBST for 1 h. The following primary antibodies were used: rabbit anti-PARP-1 (Cell Signaling, Danvers, MA) and rabbit anti-β-actin (Sigma, St. Louis, MO), diluted 1:1000 and 1:3000 with blocking solution. Anti-PARP-1 reagent detects full length PARP-1 (116 kDa), as well as its large fragment (89 kDa). Then, immunoblotted proteins labeled with primary antibody were hybridized with HRP-conjugated goat anti-rabbit (1:3000 dilution; Sigma). The mobility of the molecular weight markers is specified. Blots were treated with enhanced chemiluminescence reagent (Millipore, Billerica, MA) and exposed to x-ray films (Phenix, Candler, NC) for protein band visualization. Photographs from the developed films were captured using a gel documentation system (Alpha Innotech, San Leandro, CA).

### Polychromatic analysis of mitochondrial membrane potential (*Δ*Ψm)

Nalm-6 cells were treated with 50 µl GB and incubated for 4 h as detailed above. Then, cells were stained with 2 µM of the fluorophore 5,5',6,6'-tetrachloro-1,1',3,3'-tetraethylbenzimidazolylcarbocyanine iodide (JC-1) following the manufacturer’s instructions (Life Technologies, Grand Island, NY). The disruption of mitochondrial *ΔΨm* is evidenced by an appreciable shift of the ﬂuorescence signal from red to green. JC-1 aggregates or monomers, emitting red or green signal, were identified *via* ﬂow cytometry (Cytomics FC500) using FL2 or FL1 detectors, respectively. As a positive control a proton ionophore that dissipates the mitochondrial *ΔΨm*, carbonyl cyanide 3-chlorophenylhydrazone (CCCP, 50 µM), was used. Cells treated with the GB diluent (PBS) and untreated cells were used as controls. The data collection and analysis was performed using CXP software.

### Luminex multiplex analysis

Nalm-6 cells seeded in a multi-well plate were exposed to 25 and 50 µl/ml of GB for 3 h before the cell pellets and culture media supernatants were collected by centrifugation. Cell culture supernatants from each sample were then assayed to quantify tumor necrosis factor alpha (TNF-α) and soluble Fas ligand (sFas-L) cytokines levels, following the manufacturer’s recommendation (Millipore, Billerica, MA). In addition, cell pellets were washed with PBS and lysed with Milliplex MAP cell signaling lysis buffer containing protease inhibitors (Millipore, Billerica, MA). Protein quantifications were performed using BCA reagent (Pierce, Thermo Scientific, Rockford, IL). The MILLIPLEX MAP Human Src Family Kinase kit (Millipore, Billerica, MA) was used to detect phosphorylated Lck (Tyr394), Src (Tyr419), Fyn (Tyr420), Blk (Tyr389) and Fgr (Tyr412), in 25 µg of total cell lysate according to the manufacturer’s suggested protocol. Target proteins were analyzed using bead-based xMAP (multianalyte profiling) technology on the Luminex 200 platform (FlexMAP 3D system; Millipore, Billerica, MA) coupled with xPONENT 3.1 software (Luminex, Austin, TX) according to the manufacturer’s suggested protocol (Millipore). Each experimental point and controls were performed in triplicates.

### Real-time detection of intracellular caspase-8 activation

Caspase-8 activation in Nalm-6 cells was determined using the cell permeable and irreversibly binding caspase-8 inhibitor labeled with fluorescein, FITC-IETD-FMK, and monitored *via* flow cytometry [30]. Cells on a 24-well plate format, seeded at a density of 100,000 cells per well in 1 ml of growth culture media, were exposed to 10 and 50 µl/ml of GB for 4 h and subsequently incubated with FITC-IETD-FMK reagent, for staining purposes, following manufacturer’s protocol (abcam, Cambridge, MA). The following controls were utilized: 50 µl GB extract concomitantly added with the pan-caspase inhibitor Z-VAD-FMK (abcam) at 20 µM, which blocks the binding of FITC-IETD-FMK; 2 mM of H_2_O_2_, as a positive control for caspase-8 activation; 50 µl of PBS as solvent control; and untreated cells as a negative control. The cell distributions with green fluorescence signal were analyzed using the FL-1 detector. Data acquisition and analysis of the caspase-8-positive cells was performed using CXP software (Beckman Coulter).

### Statistical analysis

Each data point was performed a minimum three times. To indicate experimental variability, data were expressed as the average with corresponding standard deviation. Two-tailed paired Student’s *t*-tests were performed to establish the statistical significance of differences between experimental samples and their corresponding controls. To denote whether comparisons of two treatments have statistical significance, a value of *P* < 0.05 was considered significant.

## Results

### GB diminished the proliferation of leukemia/lymphoma cells

**Figure 1:**
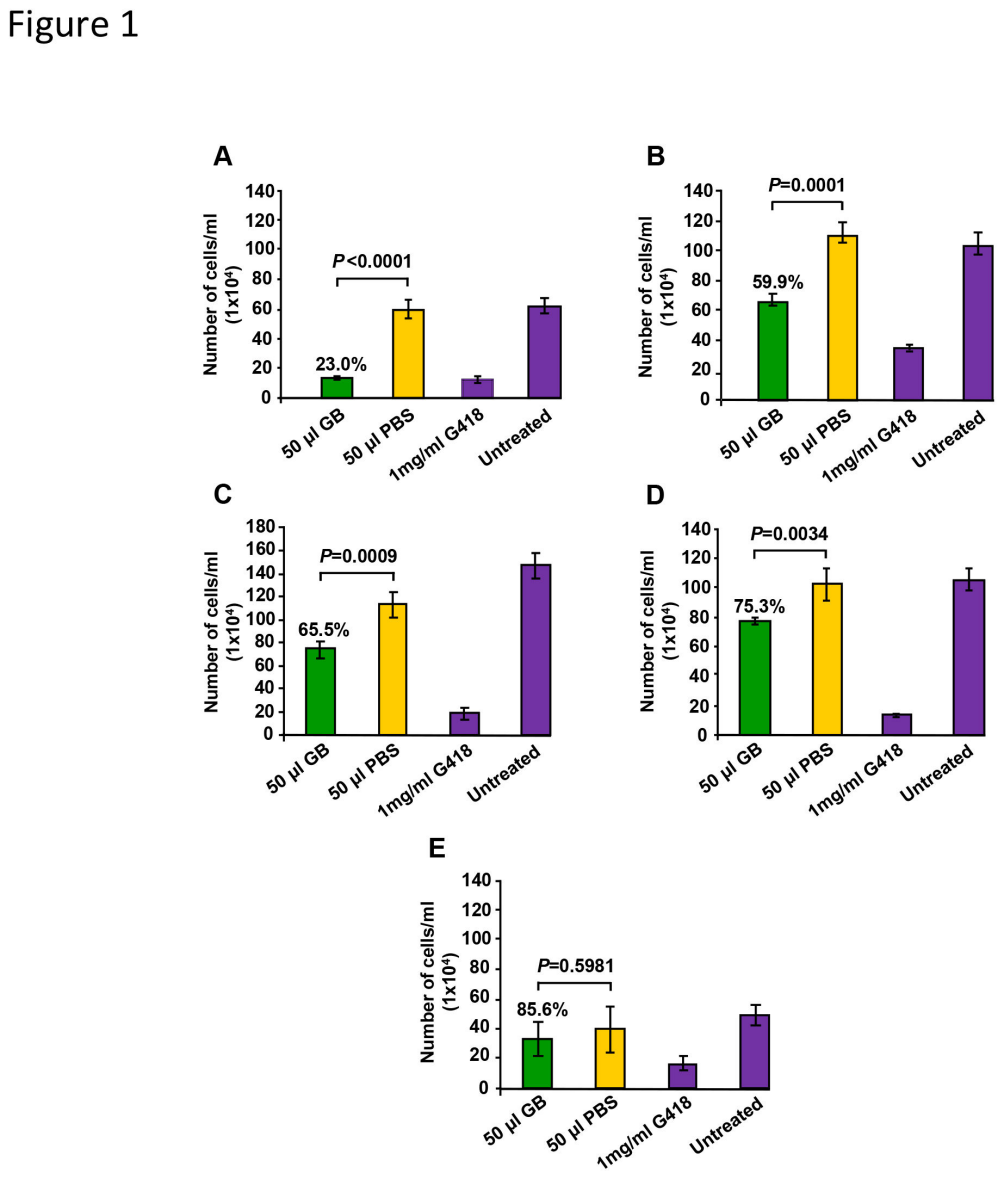
GB exhibited selective anti-proliferative activity on malignant human leukemia/lymphoma cells. The total numbers of cells per milliliter (*y*-axis) were quantified using a hemocytometer after 96 h of treatment: (A) Nalm-6, (B) BJAB, (C) YT NK-like, (D) Jurkat and (E) Hs27 cells. As a positive control of an anti-proliferative effect, 1 mg/ml G418 was included. Untreated cells were used as an indicator of cellular viability during all the incubation time periods. Additional controls of the diluent of the extracts, PBS, were also examined. Each bar represents the average of four replicates and the error bars represent the standard deviation. Cell proliferation, annotated of the top of 50 µl GB treated cells, is shown as a percentage of the cell proliferation of PBS-treated cells, which is considered 100% of growth. Fifty µl GB in PBS is equivalent to 1.5 ± 0.048 mg/ml lyophilized powder.

Initially, we examined the potential anti-proliferative activity of GB on cancer and non-cancer origin cells by quantifying the total number of cells after 96 h. GB-treated leukemia/lymphoma cells demonstrated a statistically significant reduction in their total numbers (*P* < 0.05; Figure 1). After normalization of the PBS-treated controls to 100%, the percentage of proliferation of GB-treated cells was calculated. GB exerted a gradient of inhibition of proliferation on leukemia/lymphoma cells, causing reductions in cell proliferation of 73% on Nalm-6, 40% on BJAB, 34.5% on YT and 24.7% on Jurkat cells (Figure 1A–D). Interestingly, this anti-proliferative activity did not significantly affect the proliferation of the Hs27 non-cancer cells (Figure 1E). In addition, B-lineage Nalm-6 and BJAB were the most affected cells (Figure 1A-B). Thus, these cell lines were used in further experiments to explore the mechanism underlying GB cellular toxicity. Thus far, our findings suggest that GB possesses a preferential suppression activity of leukemia/lymphoma cell proliferation.

### GB induces cytotoxicity on pre-B acute leukemia/lymphoma Nalm-6 cells

**Figure 2:**
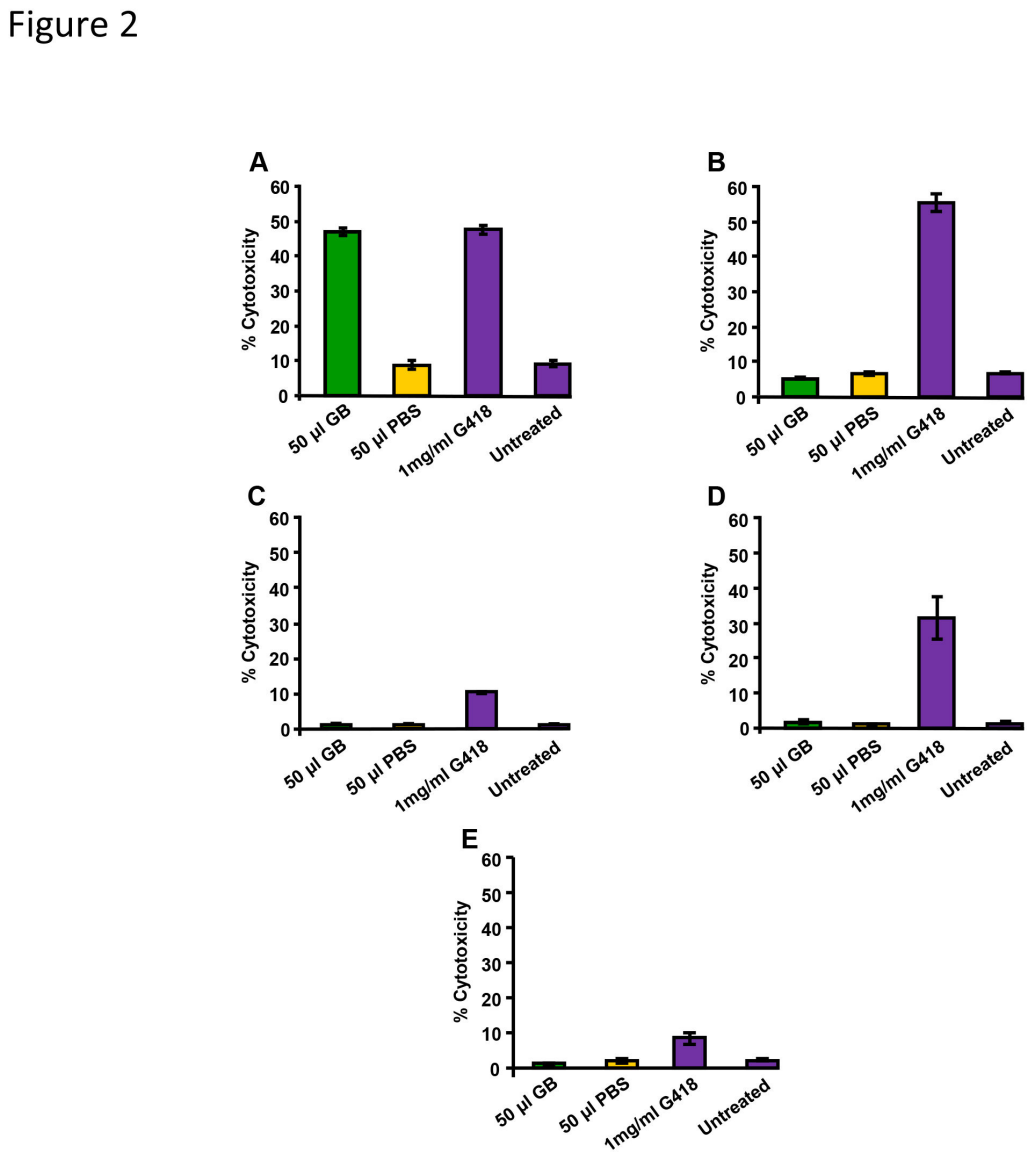
GB exerted differential cytotoxicity on leukemia/lymphoma cell lines. After 96 h of incubation, toxicity was observed on (A) Nalm-6 cells; whereas (B) BJAB, (C) YT, (D) Jurkat and (E) non-cancer Hs27 cells were resistant to this toxic effect. Cells were stained with PI and the percentage of cytotoxicity (*y*-axis) was monitored using a flow cytometric method. As a positive control of cytotoxic activity, 1 mg/ml G418 was utilized. Untreated cells were used as an indicator of cellular viability throughout the incubation period. The diluent of the GB extract, PBS, was also analyzed. Each bar represents the average of four replicates and the error bars represent the standard deviation. 1 x 10^4^ events per sample were acquired and were analyzed using CXP software (Beckman Coulter). Fifty µl GB in PBS is equivalent to 1.5 ± 0.048 mg/ml lyophilized powder.

Concomitant with the quantification of the total number of cells, we determined whether GB induced cytotoxicity. Surprisingly, cytotoxicity was observed only in Nalm-6 cells, but not in BJAB, YT, or Jurkat cells (Figure 2A–D), implying that the observed anti-proliferative activity was not necessarily associated with cytotoxicity. With the exception of Nalm-6, the viability of leukemia/lymphoma cell lines consistently resembled the values of PBS-treated or untreated controls (Figure 2). Similarly, a lack of cytotoxicity was also observed in normal non-cancer-derived cells (Figure 2E).

### GB elicits DNA fragmentation and G2/M arrest on BJAB cells

**Figure 3:**
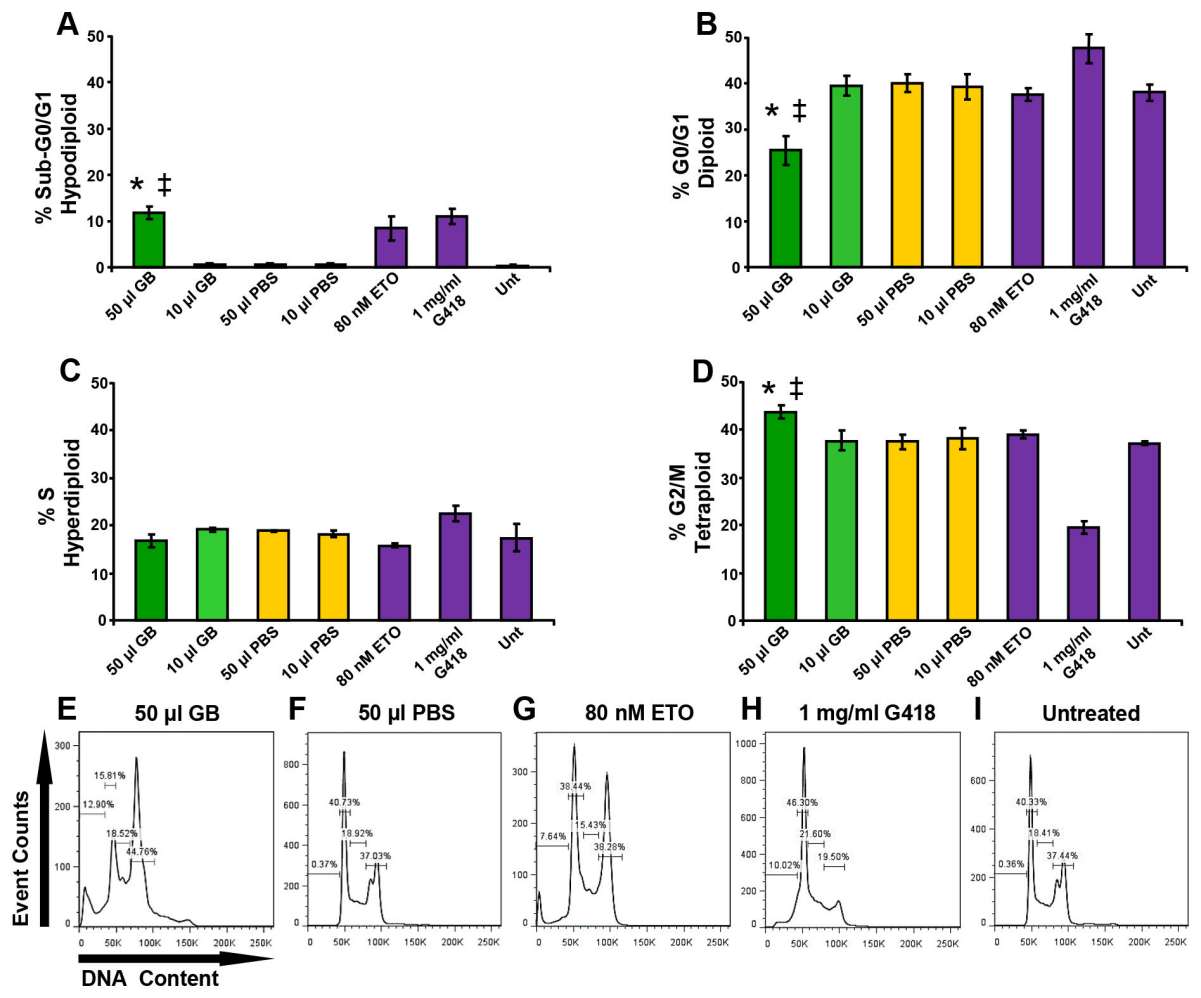
GB caused perturbation of the cell-cycle profile of BJAB cells. After 96 h GB induced apoptotic DNA fragmentation and G2/M phase arrest in a dose-dependent modality. Cells were harvested, permeabilized and stained and analyzed *via* flow cytometry. Each bar represents the average of three independent replicates, and the error bars represent the corresponding standard deviation. (A–D) The percentage of cell frequency is graphed along the *y*-axis, and the different treatments are plotted along the *x*-axis. (E–I) Representative single parameter histograms where four gates are annotated exhibiting the percentage of cell frequency in each phase of the cell cycle. Gates from left to right: sub-G0/G1 (hypodiploid; counted as an apoptotic subpopulation), G0/G1 (diploid), S (hyperdiploid) and G2/M (tetraploid). This series of experiments included several controls: two compounds provoking cell-cycle alteration, (G) 80 nM etoposide (ETO) and (H) 1 mg/ml G418; (F) the GB diluent, PBS, as contained in the experimental samples; and (I) untreated (Unt) cells, as a negative control. The significance of the differences between 50 µl GB-treated cells as compared to 50 µl PBS-treated cells, and also, with untreated cells, is of *P* < 0.03 (*) and *P* < 0.04 (‡), respectively. GB 10 µl = 0.3 ± 0.009 mg/ml, and GB 50 µl = 1.5 ± 0.048 mg/ml lyophilized powder.

Cell cycle distribution analyses were performed to decipher in more detail the mechanism that GB uses to inhibit cell proliferation. For this purpose, BJAB cells were exposed to 10 and 50 µl GB for 96 h and then prepared for analysis of cellular DNA content *via* flow cytometry. This method relies on fluorescent probes that bind DNA, thus enabling the entire DNA content per nucleus to be monitored with precision [31]. Relevant differences in cell cycle distribution were observed in cells treated with 50 µl GB, whereas 10 µl GB-treated cells did not exhibit significant alteration in the cell frequency of all cell-cycle phases (Figure 3A–D). Cells with apoptotic fragmented DNA can be distinguished by their hypodiploid DNA content (sub-G0/G1peak) during univariate cellular DNA content analysis. The hypodiploid values were increased to 11.8% in cells treated with 50 µl GB, whereas in samples exposed to 10 µl GB, PBS or untreated controls, the hypodiploid values were less than 1% (*P* = 0.01173; Figure 3). The 25.4% G0/G1 value in the GB-treated cells was significantly reduced (*P* < 0.04) compared to 40.1% and 39% for the PBS-treated and untreated cells, respectively. Differences in the frequency of cells in the S phase were imperceptible. The percentage of cells in G2/M phase for GB-treated cells was 43.7%, which was higher when compared to either the cells that were treated with PBS, 37.5%, or the untreated controls, 37.1% (*P* = 0.01945 and *P* = 0.02426, respectively).

### GB evokes phosphatidylserine externalization on B-lineage cell lines

**Figure 4:**
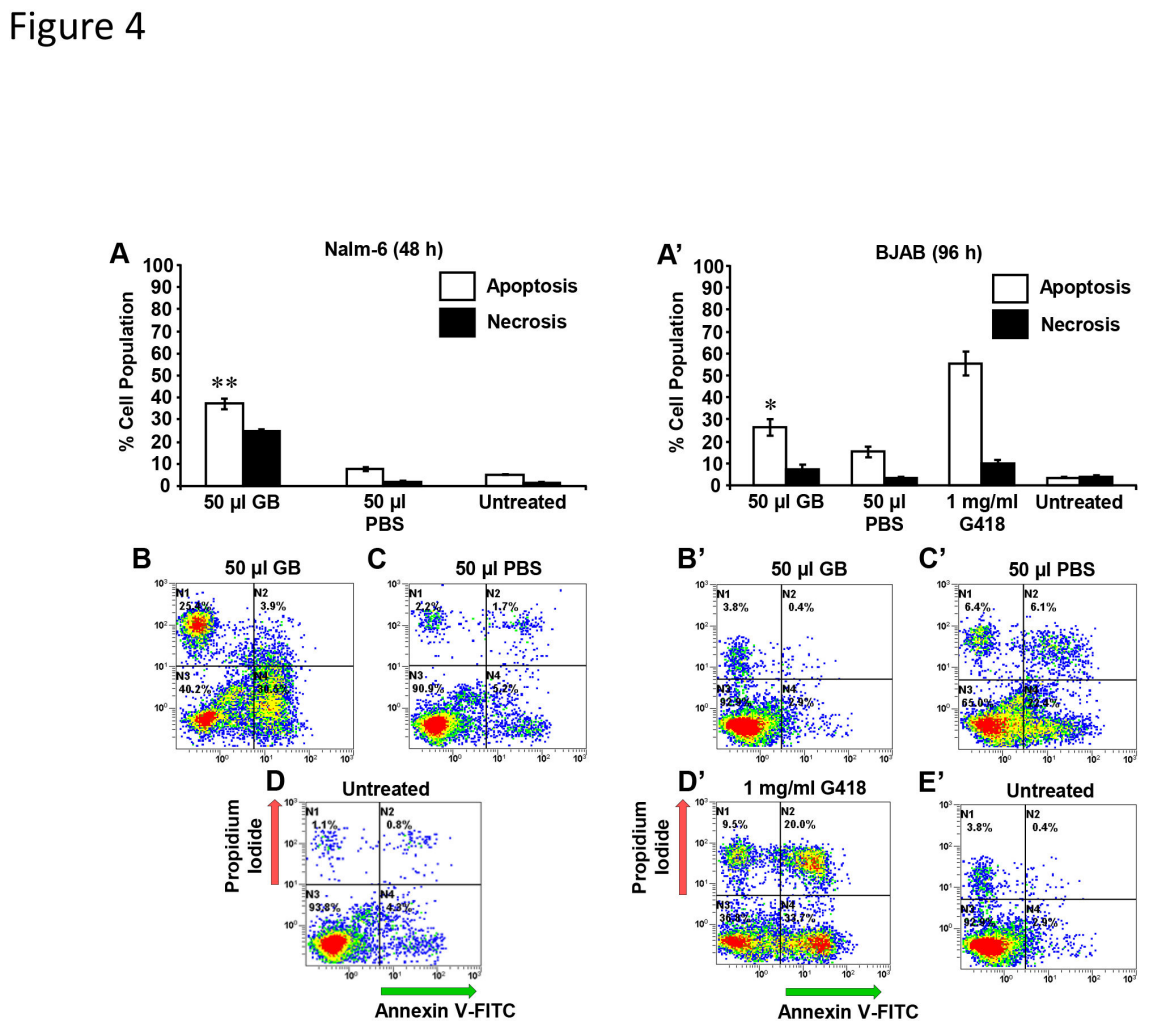
GB induced significant PS externalization on B-lymphoid lineage cells. The mode of inflicting cell death, apoptosis or necrosis, was monitored by flow cytometric assay after co-staining of the cells with annexin V-FITC and PI. Analysis was determined after 48 h and 96 h of incubation on (A–D) Nalm-6 and (A’–E’) BJAB cells, respectively. The total percentage of apoptotic cell populations is expressed as the sum of percentages of early and late stages of apoptosis (white bars), with green fluorescence signal indicating annexin V-FITC positive cells. Cells that were stained only with PI, due to the loss of plasma membrane integrity, were considered necrotic cells (black bars). Analysis of GB-treated Nalm-6 cells compared with PBS and untreated controls resulted in values of *P* < 0.00003 and *P* < 0.00002, respectively (**); whereas GB-treated BJAB cells as compared to PBS and untreated controls was *P* = 0.01284 and *P* = 0.00042 (*), respectively. Each bar represents the average of three replicas, and standard deviation. The following controls were included: cells exposed to 1 mg/ml G418, as a positive control for cytotoxicity; cells treated with 50 µl PBS; and untreated cells were also analyzed. Representative flow cytometric dot plots (A–D and B’–E’) that were used to determine the percentages of apoptosis/necrosis activity are depicted. Analysis of data from quadrant regions in the dot plots were interpreted as follows: the bottom left quadrant specifies unstained viable cells with intact membranes that are annexin V-FITC and PI double-negative; the top left quadrant denotes necrotic cells that are PI-positive and annexin V-FITC-negative; the top right quadrant includes late apoptotic cells that are annexin V-FITC and PI-positive; and the right bottom quadrant designates early apoptotic cells that are annexin V-FITC-positive, but PI-negative. The varied dot (event) color in each plot, designates just a density gradient; low-density region blue and high-density red. Approximately 1x10^4^ events were recorded and analyzed per individual sample using CXP software. GB 50 µl = 1.5 ± 0.048 mg/ml lyophilized powder.

Two B-lineage cell lines were exposed to GB to discern its potential induction of phosphatidylserine (PS) externalization. This was monitored *via* annexin V-FITC/PI assay and flow cytometry. To determine whether GB cytotoxicity exerted on Nalm-6 cells also comprised PS translocation, experiments were performed after 48 h incubation with 50 µl GB. At this time point, 37.2% of the cells were apoptotic and 24.9% of and the cells were necrotic (Figure 4A). Cells treated with 50 µl PBS and the untreated cells did not show a substantial increase in either apoptotic or necrotic values (Figure 4C-D). Additionally, the increase of apoptotic DNA fragmentation observed on GB-treated BJAB cells was further investigated under the same circumstances that were applied for the cell-cycle analysis. GB-treated cells were 26.3% annexin V-FITC positive, in contrast with 15.1 and 3.4% for PBS-treated or untreated cells, respectively; both values were significant with *P* < 0.05 (Figure 4A’-E’). Differences in the numbers of necrotic cells were essentially not detected. GB exerted PS translocation on Nalm-6 cells in a more pronounced manner than on BJAB cells (Figure 4A’). This evidence confirms that GB consistently provoked PS externalization on two B-lineage cells.

### GB-mediated cytotoxicity involves caspase-3 activation

**Figure 5:**
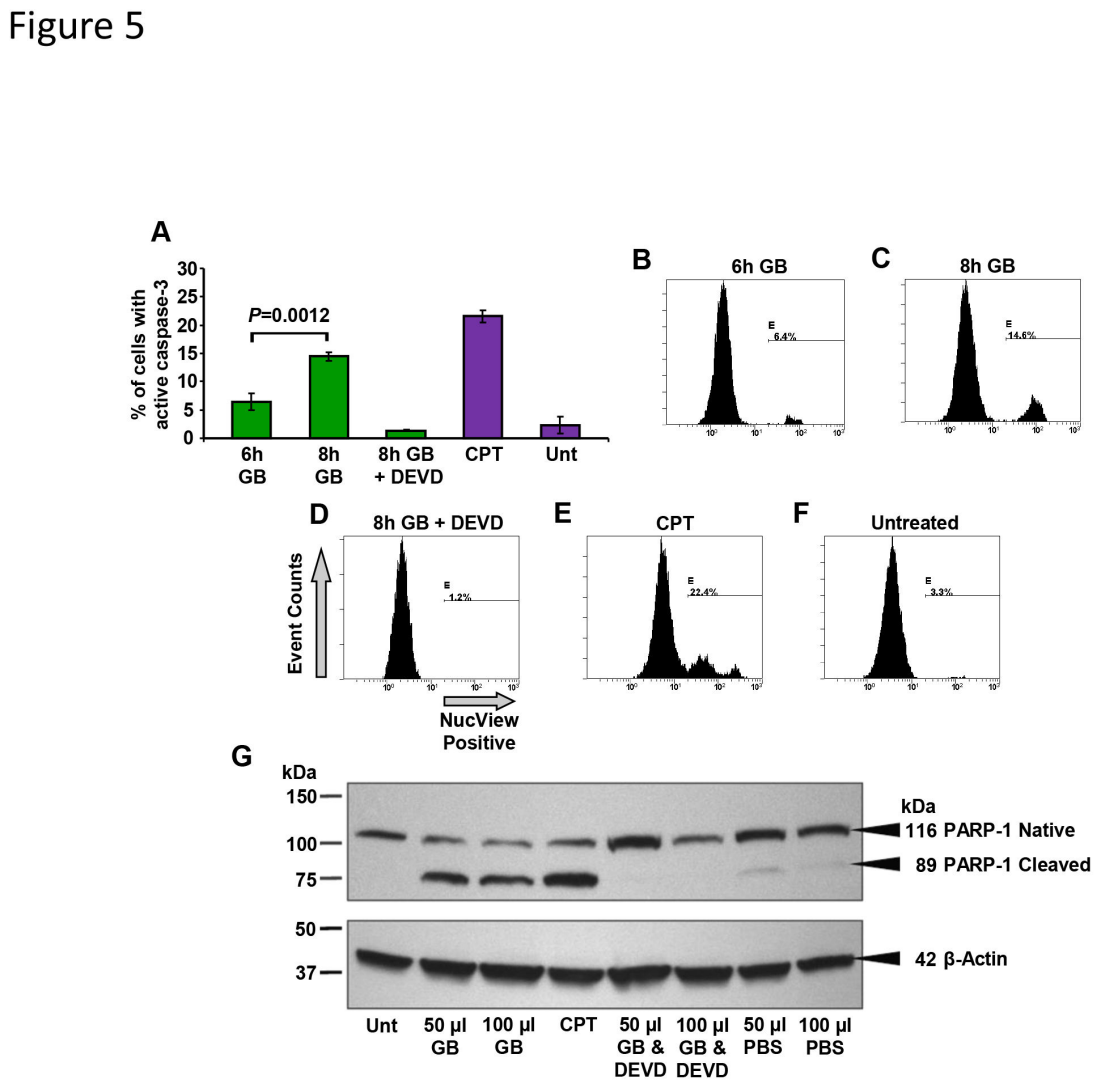
GB treatment resulted in caspase-3 activation and PARP-1 cleavage in Nalm-6 cells. Caspase -3 activation was monitored *via* (A) flow cytometry; whereas PARP-1 was analyzed *via* (G) Western blotting analysis. (A) The percentage of NucView caspase-3-positive cells exhibiting green fluorescence signal is indicated on the *y*-axis, whereas the different cell treatments are indicated on the *x*-axis. Each bar represents the average of three independent measurements, and the error bars are the corresponding standard deviations. After being exposed to GB for 6 (B) and 8 h (C), cells were incubated with a caspase-3 fluorogenic indicator substrate. (D) A set of GB-treated cells was pre-exposed to an Ac-DEVD-CHO caspase-3 inhibitor (DEVD) to add the NucView substrate. (E) As a positive control for induction of caspase-3 activation, 2 µg/ml camptothecin (CPT) was utilized. (F) Untreated cells (Unt) were used as a negative control. (B–F) Representative single-parameter histograms utilized to obtain the percentage of NucView caspase-3 substrate-positive cells: the number of event counts (cells) is plotted on the *y*-axis, whereas the measurement parameter, cells with green fluorescence signal NuvView positives, is plotted on the *x*-axis. Approximately, 1x10^4^ events were acquired and analyzed per sample using CXP software. (**G**) **Full-length PARP-1 (116 kDa) and its large fragment (89 kDa) were analyzed on GB-treated Nalm-6 cells after 24 h**. As a positive control for PARP-1 cleavage, 2 µg/ml camptothecin was used. Where indicated, some cells were incubated concurrently with GB and the caspase-3 inhibitor DEVD. β-actin was used as a housekeeping protein loading control (bottom panel). The mobility of the protein molecular weight markers is specified on the left. GB 50 µl = 1.5 ± 0.048 mg/ml, and GB 100 µl = 3.0 ± 0.009 mg/ml lyophilized powder.

To determine if caspase-3 activation was directly involved during GB cytotoxicity, Nalm-6 cells and a cell permeable fluorogenic substrate were utilized. The substrate facilitates the detection of caspase-3/7 activity in intact cells *via* flow cytometry. Caspase-3 activation was detectable as early as 6 h and increased significantly at 8 h (*P* = 0.0012) on GB-treated cells (Figure 5A). Additionally, a highly specific and potent inhibitor of casapse-3, Ac-DEVD-CHO, was selected to confirm whether the method utilized to detect caspase-3 activation was a specific biochemical reaction [32]. This unlabeled tetrapeptide aldehyde encompasses the amino acid motif of the PARP-1 cleavage site and competes with the active site of caspase-3 [32]. Cells were exposed to GB in concurrence with the unlabeled Ac-DEVD-CHO caspase-3 inhibitor, resulting in an important reduction of caspase-3 activation (Figure 5D). These observations suggest that GB has a propensity to induce caspase-3 activation in a time-dependent fashion on Nalm-6 cells, and this activation was minimized by the caspase-3 inhibitor.

### GB provokes PARP-1 cleavage

Activation of PARP-1 expedites cellular disassembly by rapid catalysis of NAD^+^ and subsequent ATP depletion, and it also serves as a robust indicator of cells undergoing apoptosis [33,34]. Consequently, we tested whether GB-treated Nalm-6 cells exhibited PARP-1 cleavage, as visualized by Western blotting. PARP-1 cleavage was clearly observed on GB-treated cells, as evidenced by the presence of an 89 kDa fragment (Figure 5G). Again, to confirm the specificity of this reaction, cells were exposed to GB along with the unlabeled Ac-DEVD-CHO caspase-3 inhibitor, which eliminated PARP-1 cleavage (Figure 5G). These results suggest that GB caused PARP-1 cleavage and that this proteolytic reaction was substantially reduced in the presence of the caspase-3 inhibitor. Indeed, this supports the conclusion that caspase-3 activation was required for PARP-1 cleavage on GB-treated Nalm-6 cells.

### GB-inflicted cytotoxicity uses a mitochondrial-independent pathway

**Figure 6:**
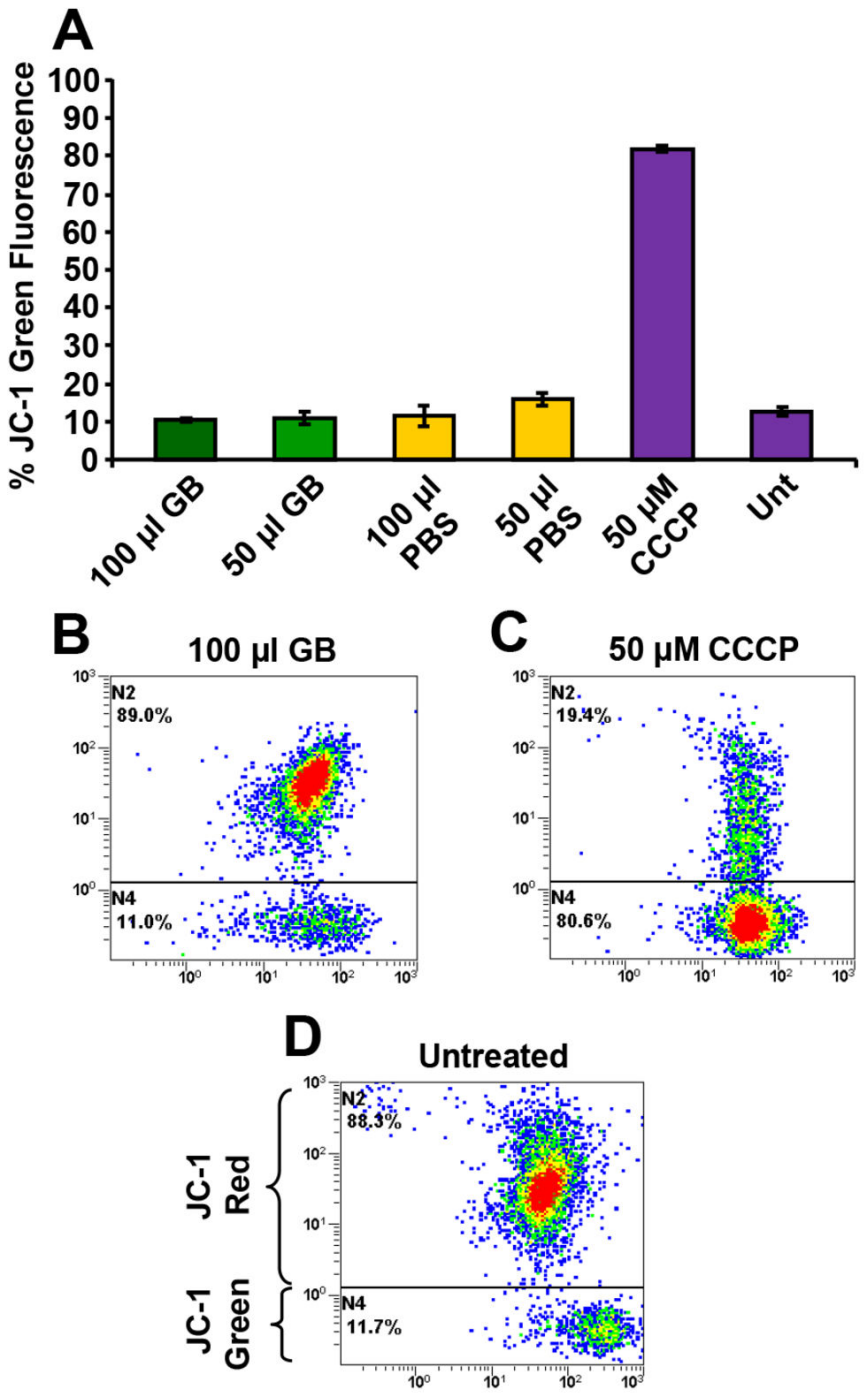
GB-mediated cytotoxicity is independent of mitochondrial *ΔΨm* disruption in Nalm-6 cells. Cells were exposed for 4 h to GB and detection of changes in the mitochondrial *ΔΨm* was determined by staining with the aggregate-forming lipophilic cationic fluorophore JC-1. After dissipation of mitochondrial *ΔΨm*, the JC-1 reagent emitted a green fluorescence signal, whereas cells with polarized mitochondrial membrane emitted a red signal. (A) Percentages of cells emitting green fluorescence signal (*y*-axis) versus treatment type (*x*-axis) are depicted. Each bar represents the average of four replicates, and the error bars represent the standard deviation. (B–C) Representative flow cytometric dot plots used to quantify the percentages of green and red signals. (C) Cells exposed to the mitochondrial uncoupler carbonyl cyanide m-chlorophenylhydrazone (CCCP; 50 µM) were used as positive controls. PBS solvent controls and untreated cells were included. The different dot (event) color in each plot, designates just a density gradient; low-density region blue and high-density red. Approximately 1x10^4^ events were acquired and analyzed per sample using CXP software. GB 50 µl = 1.5 ± 0.048 mg/ml, and GB 100 µl = 3.0 ± 0.009 mg/ml lyophilized powder.

Cells exposed to an aggressor using an intrinsic pathway and labeled with a JC-1 reagent should exhibit a green fluorescence signal, which indicates the dissipation of mitochondrial *ΔΨm*. This event should precede caspase-3 activation [35]. Therefore, we investigated whether GB was predominantly using the intrinsic pathway to evoke its death-inducing signal after 4 h of treatment. As depicted in Figure 6, cells exposed to GB (Figure 6B) resemble the profile distribution of untreated cells (negative control; Figure 6D), suggesting that GB does not interfere with the formation of JC-1 aggregates (red signal; Figure 6), which is indicative of unharmed mitochondria. A shift in the chromatic signal from red to green was only detected in the CCCP-treated positive control samples (Figure 6C). These outcomes suggest that GB-mediated cytotoxicity appeared to bypass mitochondrial *ΔΨm* perturbation.

### GB induces TNF-α release in Nalm-6 cells

**Figure 7:**
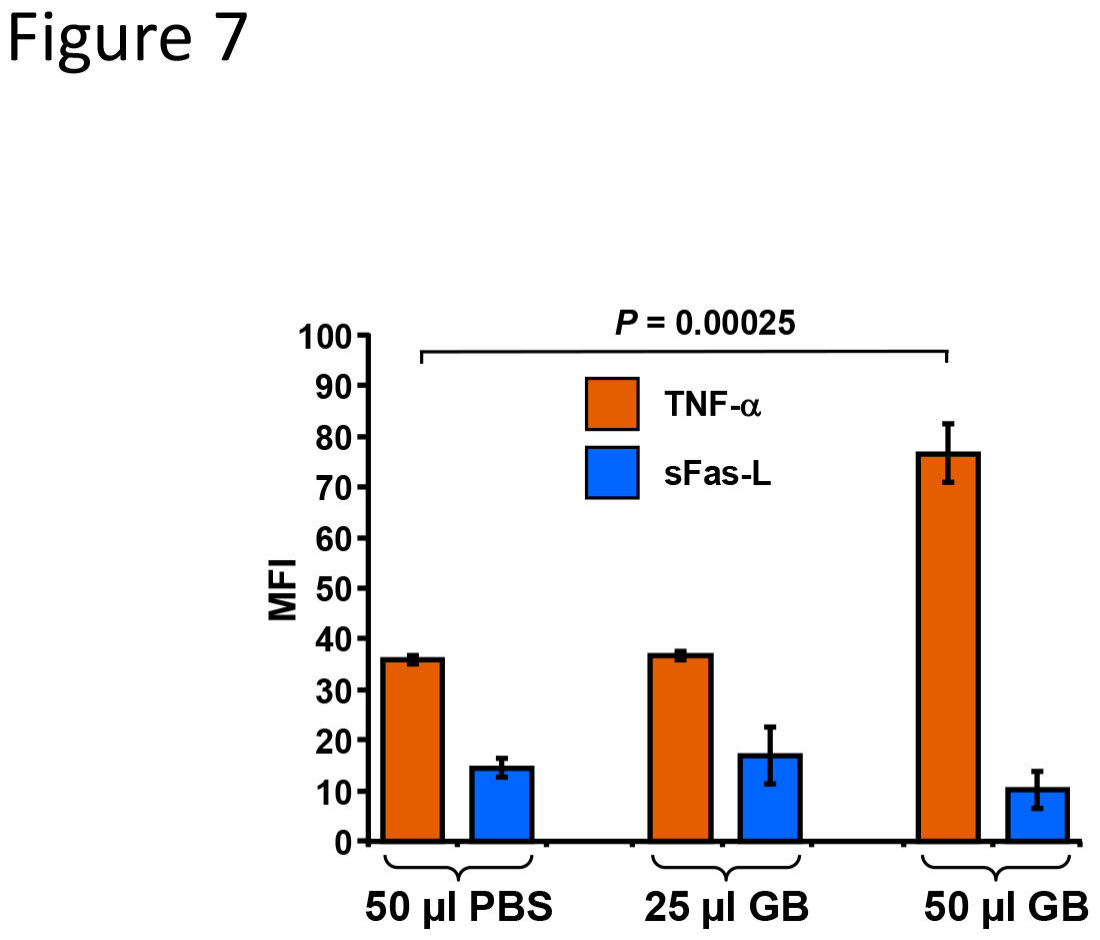
GB treatment of Nalm-6 promotes TNF-α production. Supernatants from cells exposed to 25 µl and 50 µl/ml of GB were analyzed for the presence of TNF-α and sFas-L cytokines using a bead-based Luminex xMAP platform. Cells exposed to 50 µl of PBS, solvent control, were analyzed concurrently. In the *y*-axis is annotated median fluorescence intensity (MFI); whereas in the *x*-axis the diverse treatments. Each bar represents the average and standard deviation of triplicate wells. Statistical significance was determined using Student’s *t*-test (*P*= 0.00025). Data acquisition and analysis were performed using xPONENT 3.1 software (Luminex).

In an effort to investigate the extrinsic apoptotic mechanism exerted by GB, TNF-α and sFas-L levels were examined in the supernatant of Nalm-6 cells treated for 3h with increasing amounts of GB or PBS control. Supernatants of cells treated with 25 µl of GB exhibited similar amounts of TNF-α compared with solvent control (PBS; Figure 7). However, compared to the PBS treated controls cells, treatment with 50 µl GB resulted in a significant increase in the presence of TNF-α (35.8 to 76.6 MFI; Figure 7). sFas-L levels were not affected by GB treatment (Figure 7). These results suggest that the cytotoxic effect of GB could be initiated *via* a TNF death receptor (TNF-R) pathway, thus further implicating an extrinsic apoptotic pathway as GB’s mechanism of action.

### GB-mediated cytotoxicity implicates caspase-8 activation

**Figure 8:**
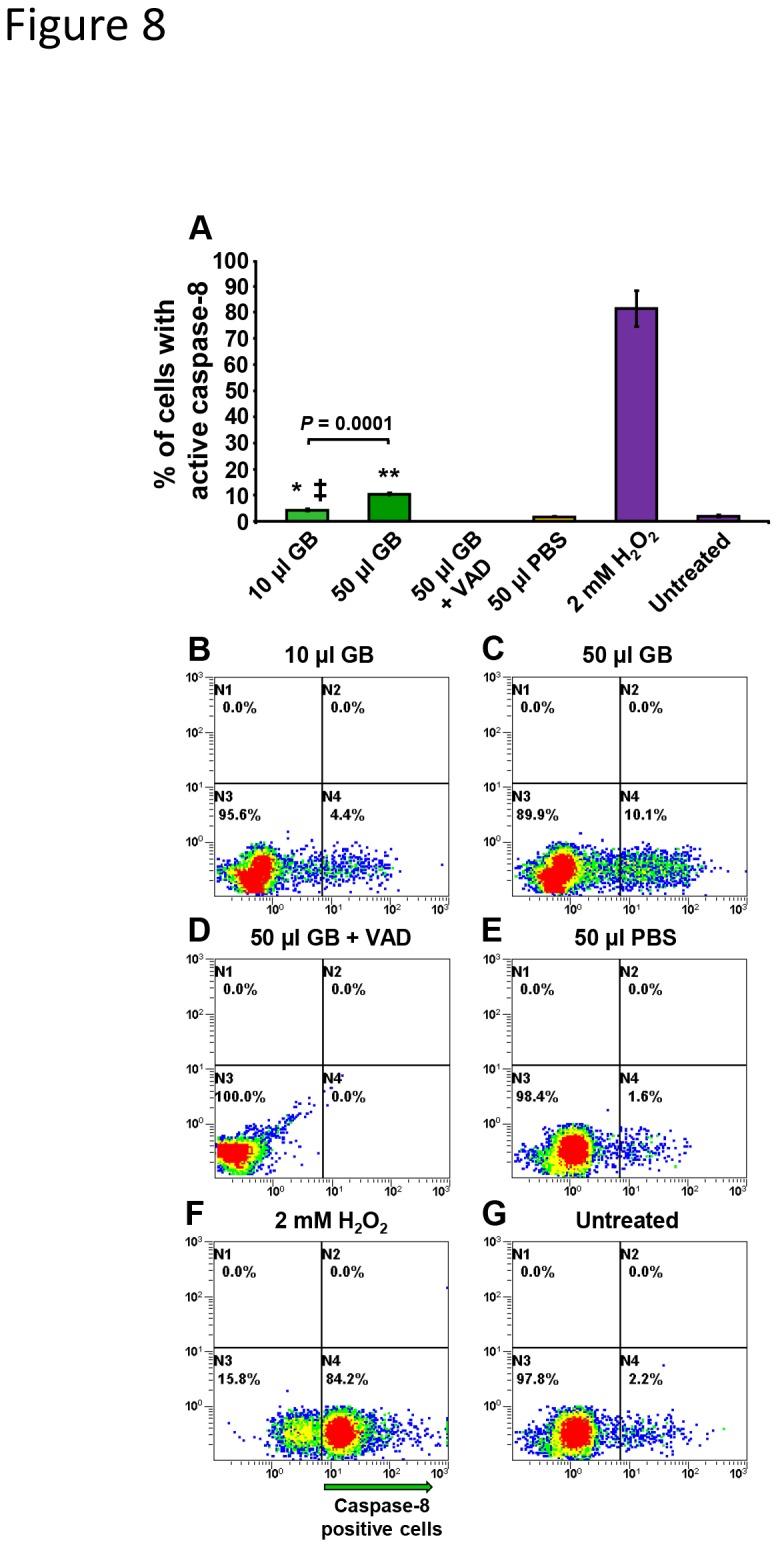
Dose-dependent activation of caspase-8 pathway by GB in Nalm-6 cells monitored *via* flow cytometry. (A) After 4 h of GB treatment, the percentage of caspase-8-positive cells exhibiting green fluorescence signal is indicated on the *y*-axis, whereas the different cell treatments are indicated on the *x*-axis. Each bar represents the average of three independent measurements, and the error bars are their corresponding standard deviations. Representative flow cytometric dot plots (B–G) that were used to determine the distribution of caspase-8-positive cells are depicted. The diverse dot (event) color in each plot, designates just a density gradient; low-density region blue and high-density red. The flow cytometer acquisition settings were as following: FL1 and FL2 detectors were plotted at *x*-axis versus *y*-axis, respectively. Cells were exposed to 10 µl (B) and 50 µl (C) of GB for 4h and then, stained with FITC-IETD-FMK, as detailed in Materials and methods; this bar is imperceptible due a low value (<0.1%). (D) A set of GB-treated cells was concurrently exposed to 50 µl of GB and 20 µM of Z-VAD-FMK cell-permeant pan-caspase inhibitor (VAD). (E) PBS solvent control, where an error bar 0.11% is not noticeable and (G) untreated cells were included. (F) As a positive control for induction of caspase-8 activation, 2 mM of H_2_O_2_ was utilized. Approximately, 1x10^4^ events were acquired and analyzed per sample using CXP software. GB 10 µl = 0.3 ± 0.009 mg/ml, and GB 50 µl = 1.5 ±0.048 mg/ml lyophilized powder. The significance of the differences between 10 µl GB-treated cells as compared to 50 µl PBS-treated cells, and also, with untreated cells, is of *P* = 0.00039 (*) and *P* = 0.00203 (‡), respectively; whereas 50 µl GB-treated cells as compared with 50 µl PBS-treated cells was consistently *P*<0.0001(**) in both data sets. GB 10 µl = 0.3 ± 0.009 mg/ml, and GB 50 µl = 1.5 ± 0.048 mg/ml lyophilized powder.

To examine whether GB cytotoxicity involves the participation of caspase-8 activation in Nalm-6 cells, a cell-permeable fluorescently conjugated (FITC) caspase-8 inhibitor and live cell-based flow cytometry assay were utilized. Caspase-8 activation was identified as early as 4 h in 10 µl GB-treated cells and increased significantly as compared with 50 µl GB-treated cells (*P* = 0.0001; Figure 8A-C). Additionally, when cells were simultaneously exposed to both 50 µl of GB and 20 µM of Z-VAD-FMK, a non-fluorescence broad-spectrum caspase inhibitor, the percentage of stained cells with green fluorescence signal was completely abolished (<0.1%; Figure 8A and D), confirming that FITC-IETD-FMK caspase-8 inhibitor specifically labels cells with activated caspase-8. Hydrogen peroxide induces apoptosis or necrosis, at low or high concentration, respectively. We utilized high concentrations of H_2_O_2_ (2 mM) to induce toxicity [36] (Figure 8A and F) and as a caspase-8 activator. Consistently, solvent and untreated controls showed similar low levels of active cellular caspase-8; 1.6%±0.11 and 2.1%±0.36, respectively (Figure 8A, E and G). Taken together these findings indicate that GB promotes caspase-8 dependent apoptosis in a dose-dependent manner in Nalm-6 cells *via* an extrinsic apoptotic mechanism.

### GB enhances Lck and Src tyrosine kinases phosphorylation

**Figure 9:**
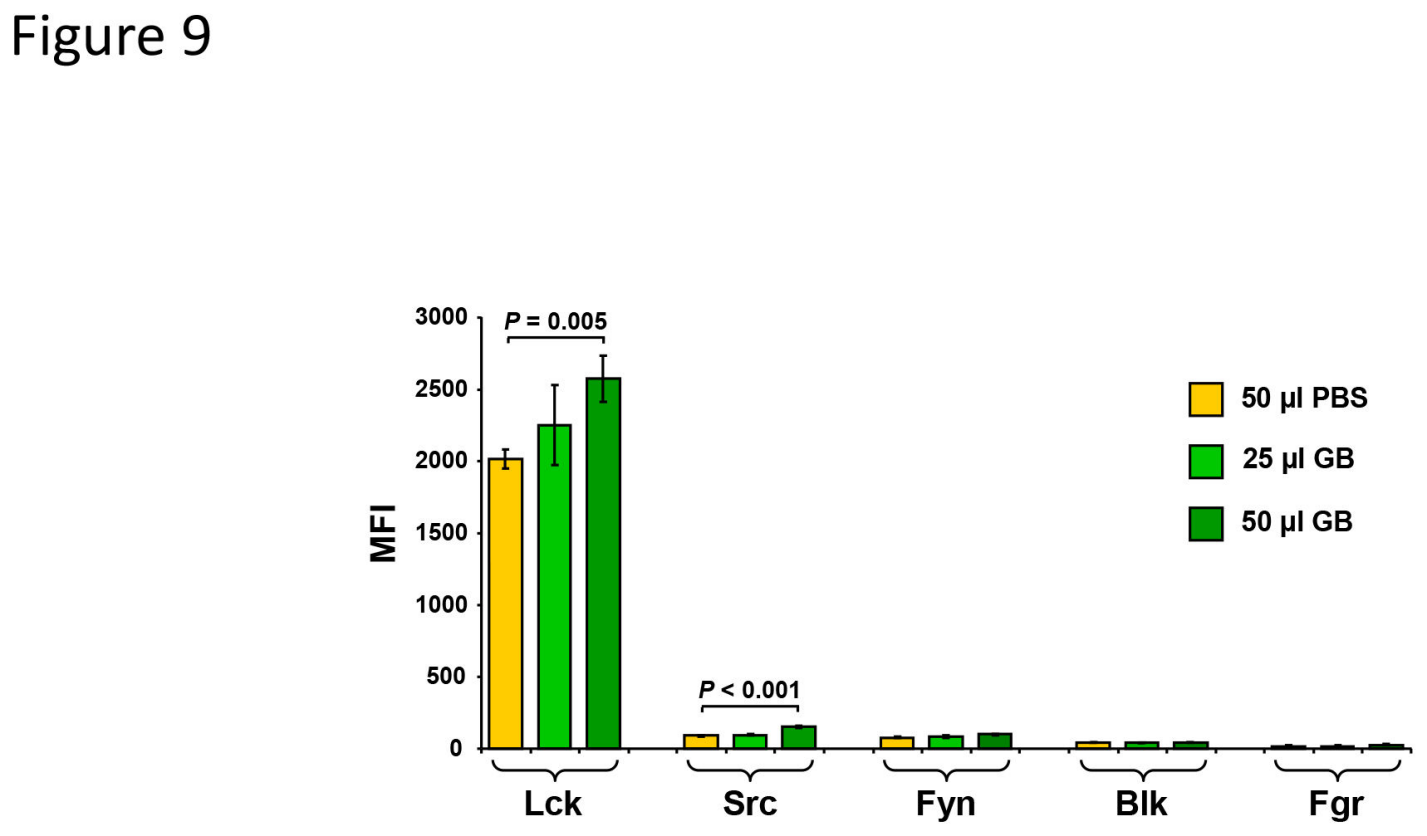
GB treatment of Nalm-6 cells results in activation of Lck and Src tyrosine kinases. Cellular extracts from Nalm-6 cells treated with 25 and 50 µl/ml of GB were analyzed for SFK activation using the bead-based Luminex xMAP platform. Cells treated with 50 µl of PBS control were analyzed in parallel. In the *y*-axis is plotted the median fluorescence intensity (MFI); whereas in the *x*-axis the different Src family kinase. Each bar represents the average of triplicates, and the error bars are their corresponding standard deviations. Statistical significance was determined using Student’s *t*-test. Data acquisition and analysis were performed using xPONENT 3.1 software (Luminex).

Cell signaling analysis of a panel of protein tyrosine kinases was performed on GB-treated Nalm-6, after 3 h of exposure to several GB concentrations, using bead-based multiplex technology. Comparison between cells exposed to 25 µl of GB with solvent control (PBS) cells, displayed minor if ant differences in expression of Lck and Src kinases (Figure 9). Nevertheless, under the same conditions, cells treated with 50 µl of GB exhibited a significant increase in expression of Lck and Src kinases, with *P*=0,005 and *P*<0.001, respectively. On the other hand, Fyn, Blk and Fgr enzymes did not show change is expression in GB treated Nalm-6 cells at any concentration studied (Figure 9). The results showed that GB was able to induce significant up-regulation of Lck and Src tyrosine kinases phosphorylation in a dose-dependent fashion, suggesting their possible contributions to a pro-apoptotic pathway.

## Discussion

In the present study, we tested an aqueous green barley extract (GB) for its potential to exert selective inhibition of cell proliferation *in vitro*, using a panel of human leukemia/lymphoma cell lines. Additionally, the possible pathways activated by GB-mediated cytotoxicity and the implicated molecular regulators have been investigated. For comparison purposes, one human non-cancer origin line was included. Initially, the absolute numbers of GB-exposed cells were quantified after 96 h of incubation. Findings exhibited that GB possess preferential and significant suppression of proliferation on all leukemia/lymphoma cells that were tested. In contrast, non-transformed human foreskin fibroblast cells (Hs27) were not significantly affected (Figure 1). In this series of experiments, B-lineage leukemia/lymphoma cells (Nalm-6 and BJAB) were the most affected by GB’s anti-proliferative activity; thus, they were selected to elucidate and characterized the biochemical machinery that GB uses to cause cytotoxicity.

Independently of the apoptotic pathway utilized to initiate cytotoxicity, cell death progression will eventually conclude with the loss of plasma membrane integrity, which is a commonly accepted criterion to denote cell death. Dead and live cell frequency values were assessed with the use of fluorescent exclusion dye PI and flow cytometry. After 96 h of exposure to GB (50 µl), the cell death values obtained from YT NK-like, Jurkat, and BJAB cells resembled the values from untreated cells (Figure 1), which indicated that GB acted as suppressor of proliferation on the leukemia/lymphoma cells, without inflicting cell death. Conversely, under the same experimental conditions, pre-B ALL cells revealed marked cytotoxicity (~ five-fold compared with PBS treated cells) that was associated with suppression of cell growth. This cell line was the most sensitive to GB treatment. Our observation was that GB inhibited proliferation of the four leukemia/lymphoma cells and this activity was only associated with cytotoxicity on Nalm-6 cells. Hence, GB caused differential toxicity on leukemia/lymphoma cells, concomitant to anti-proliferation activity. Moreover, GB did not significantly affect proliferation or viability on cells from non-cancer origin (Hs27).

In the apoptosis process, one of the multiple biochemical alterations is cellular DNA fragmentation that is provoked by endogenous caspase-activated deoxyribonuclease and consequently in the generation of diffusible small fragments from the cell that is experiencing apoptosis [37]. Cell-cycle analyses were performed to determine whether the suppression of proliferative effect of GB was associated with uncoupling the cell-cycle progression profile and the possible induction of DNA fragmentation, as assessed by flow cytometry. For this purpose, BJAB cells were selected on the basis that they did not exhibit noticeable cytotoxicity, even after prolonged exposure to GB (96 h). The monitoring strategy was to label cells with a fluorescent nucleic acid intercalator [38]. Thus, cells emit a fluorescence signal that is directly proportional to their DNA content [31]. Using this type of analysis, various distinct facets of the cell cycle are recognized, but some of them are very difficult to separate based on DNA content and PI staining; G0 is indistinguishable from the G1 phase, and similarly, G2 from the M phase, because both have an identical DNA content. Hence, in a given normal proliferating cell population, three populations are generally detected by flow cytometry: G0/G1, one set of paired chromosomes per cell, diploid (2n); S, DNA synthesis with variable amount of DNA, going from diploid to tetraploid (between 2n and 4n); and G2/M, two sets of paired chromosomes per cell, prior to cell division, tetraploid [39]. Furthermore, apoptotic cells are also relatively easily identified because they manifest a decrease in total fluorescence intensity; some small fragments diffuse out of cells (or nucleus), resulting in cells containing less diploid DNA content (< 2n; sub-diploid), generating a definable population with a sub-G0/G1 peak [40]. In each experiment, two cell-cycle disruptors were utilized: G418, which blocks protein synthesis in eukaryotic cells [41] and has therefore been inferred to arrest cells in G0/G1 stage; and etoposide, which causes G2/M phase arrest on lymphoma cells, including BJAB cells [42]. Previous reports have shown that phytochemical extracts from cranberries [43] and garlic [44] induced anti-proliferation and are associated with significant arrest at the G0/G1 phase on prostate cancer LNCaP cells and the G2/M phase on MDA-MB-435 cells. However, to the best of our knowledge, there is no evidence of GB’s effect on the cell cycle distribution in leukemia/lymphoma cells. GB-treated BJAB cells exhibited bifurcated alteration of the cell-cycle profiles in a dose-dependent manner, as displayed by the sub-G0/G1 (hypodiploid) phase increment that is indicative of DNA fragmentation, a late apoptotic event [45], and G2/M phase arrest (Figure 3). This cell-cycle distribution profile emulated to some extent the pattern that was generated for etoposide (Figure 3G). Similar apoptotic DNA damage-induced apoptosis results were obtained when BJAB cells were exposed to polyhydroxystilbenes: resveratrol found at high concentrations in grape berry skins and its hydroxylated analog, piceatannol, from *Euphorbia* *lagascae* seeds [46].

Phosphatidylserine is preferentially located in the inner leaflet of the plasma membrane and when is externalized to the outer leaflet, serves as a sensitive marker of cells undergoing apoptosis [47]. Detailed studies have revealed that phytochemicals, such as an organosulfur compound obtained from garlic, ajoene [48], and a phloroglucinol compound from St. John’s wort, hyperforin [49], exert pro-apoptotic activity, as evidenced by PS externalization in acute myeloid leukemia HL-60 cells or B-cell chronic lymphocytic leukemia cells from patients, respectively. To further validate the observation whether DNA-fragmentation occurrence was an apoptotic event, which was detected in BJAB cells by quantiﬁcation of sub-G0/G1 fraction through cell cycle analysis (Figure 3), we examined if GB was able to perturb plasma membrane phospholipid asymmetry. In a series of experiments, cells were dual stained with annexin V-FITC and PI to analyze the PS translocation. This assay was performed on Nalm-6 cells in conjunction with its more differentiated BJAB cells; both are related to the B-lymphoid lineage. Nalm-6 cells exposed to GB exhibited a higher number (37.2%) of annexin V-FITC positive cells after 48 h. Additionally, under the same circumstances employed during the DNA content cell-cycle analysis of GB-exposed BJAB cells (96 h), the number of annexin V-FITC positive cells was increased to 26.3% (*P* = 0.01173; Figure 4). In agreement with those findings, GB consistently stimulated PS externalization, which was more pronounced on Nalm-6 than BJAB cells. Moreover, the DNA fragmentation provoked by GB on BJAB cells correlated with PS translocation, suggesting that both events are apoptosis-related biochemical characteristics.

Although two main distinct signal pathways can trigger programed cell death, intrinsic/mitochondrial and extrinsic/death receptor, the middle sequential execution facets are highly conserved [50]. Previous report indicated that vernodalin, a phytochemical compound isolated from *Centratherum* *anthelminticum* (L.) seeds, inhibited growth of human breast cancer cells stimulating apoptosis *via* caspase-3 activation [15]. We next examined the influence of GB on two of the pivotal effectors in pro-apoptosis, caspase-3 and PARP-1, on Nalm-6 cells. Activation of caspase-3 is the convergence point of both upstream apoptotic pathways [16] and is an early occurring event, even prior to the appearance of morphological characteristic changes to identify cells experiencing apoptosis [17]. Caspase-3 is synthesized as an inactive 32 kDa proenzyme that is proteolytically cleaved [51], generating two subunits of 17 kDa and 20 kDa that, once heterodimerized, constitute the functionally active enzyme [50]. Our study showed a significant increase of caspase-3 activation in GB-treated Nalm-6 cells at 6 and 8 h. In addition, when a caspase-3 inhibitor (Ac-DEVD-CHO) was added to the assay, caspase-3 activation was abolished. These findings suggest that GB can induce activation of caspase-3 in Nalm-6 cells, as its mode to exert cell death. Moreover, this activation appears to be in a time-dependent modality.

As essential protagonist in the execution of apoptosis-mediated cell death, caspase-3 is the principal proteinase leading to the proteolytic cleavage of PARP-1, its downstream target nuclear substrate [32,52]. PARP-1 is a DNA repair enzyme that is responsible for preserving the genomic integrity, and its cleavage is a pro-apoptotic signature [53]. Activation of PARP-1 expedites cellular disassembly and also serves as a strong indicator of cells undergoing apoptosis [33,34]. Excessive PARP-1 activation, in response to massive DNA damage, signals the depletion of NAD^+^ (a PARP-1 substrate) and ATP, provoking a dramatic reduction of cellular energetic pools, which is a typical occurrence in the progression of apoptosis [54], culminating in cellular dysfunction and death [55]. Our data show that PARP-1 cleavage occurred in Nalm-6 cells exposed to GB, and this reaction was abrogated by Ac-DEVD-CHO, suggesting that caspase-3 was necessary for GB-induced PARP-1 cleavage. In this context, the treatment of cells with a caspase-3 inhibitor conspicuously prevented both the caspase-3 activity, as well as PARP-1 cleavage. These data suggest that GB induces apoptosis in the Nalm-6 cells by eliciting a caspase-3 activation →PARP-1 cleavage cascade, a central apoptotic signaling pathway.

When mitochondria are used to initiate apoptosis (intrinsic pathway), its *ΔΨm* is disrupted during the early stages of the program [56], occurring before caspase-3 activation. Conversely, if any compound is not using the intrinsic pathway, mitochondrial *ΔΨm* is maintained intact in the early phases of apoptosis, but should be secondarily dissipated as a influence of DNA fragmentation and ATP depletion during the final stages of apoptosis [57]. It was reported that some plant origin flavonoid (polyphenol) derivatives had no influences on the mitochondrial complex (I to V) activity of rat liver mitochondria, demonstrating that the mitochondrial *ΔΨm* is not disrupted [58]. In contrast, aqueous cinnamon extract ( *Cinnamomum* *cassia* ) induced apoptosis in a human cervical cancer cell line (SiHa) through the loss of mitochondrial *ΔΨm* [59]. In an attempt to discern what initial signal GB was using to trigger cytotoxicity, the JC-1 reagent and Nalm-6 cells were utilized. Consistently, cells exposed to GB mirrored the untreated controls, exhibiting an absence of green signal, revealing intact mitochondrial *ΔΨm*. Thus, GB-induced cytotoxicity presumed to be independent of mitochondrial *ΔΨm* perturbation.

We demonstrated that GB treatment was unable to dissipate mitochondrial *ΔΨm* in Nalm-6 cells, therefore it was hypothesized that GB exerts its cytotoxic effect through activation of an extrinsic apoptotic pathway. To test this hypothesis, TNF-α and sFas-L levels and caspase-8 activation status was examined in GB-treated Nalm-6 cells. TNF-α is a crucial protagonist for diverse cellular responses, ranging from inflammation, cell survival, and cell death [60]. TNF-α is produced by numerous cell types, including lymphoid cells [61]. Upon binding to its cognate receptor (TNF-R), the formation of the death inducing complex is initiated, which consists of receptor interacting protein kinase (RIPK1), Fas-Associated protein with Death Domain (FADD) and procaspase-8, forming the main TNF-α canonical pathway [62]. Previously, it was reported that fractions of GB extract incubated for 24 h with human monocytes THP-1 cell line, in combination with lipopolysaccharide (LPS) stimulation for an additional 5 h, exhibited a decrease in TNF-α production/release [4]. Contrary to this report, we show that Nalm-6 cells exhibited significant increase (*P* = 0.00025) in release of TNF-α as early as 3 h post treatment with GB. These findings suggest that GB exerts its cytotoxic effect on leukemia/lymphoma cells through an extrinsic apoptotic pathway, and the effect of GB on TNF-α production is cell type specific.

Next, we explored whether the increase in of TNF-α levels resulted in activation of the caspase-8 enzyme. Caspase-8 is an effector protein that plays an essential role in the extrinsic apoptotic cascade, and is initiated through upstream activation of the death receptor complex (TNF-α -TNF-R) [63]. The major downstream cellular substrate of caspase-8 is pro-caspase-3, which after proteolytic cleavage becomes an active protease [64] and is a central executioner for the progression of both extrinsic and intrinsic programs. Previously, it was demonstrated that dandelion ( *Taraxacum* *officinale* ) aqueous extracts, used in traditional medicine for treatment of leukemia and breast cancer, exhibited activation of caspase-8 in human leukemia cells [65]. Indeed, in the present study we show that GB-treated Nalm-6 cells exhibited activation of caspase-8 in a dose-dependent manner. Together these findings support the role of TNF-α in GB induced cell death of leukemia/lymphoma cells.

In an effort to understand the intracellular series of events elicited by GB, we investigated the ability of GB to modulate the activation of Src family tyrosine kinases (SFK) in Nalm-6 cells. It is well established that activation of SFKs play a pivotal role in mitogenesis through promotion of lymphocyte activation and proliferation [66]. Conversely, the SFK Lck has also been shown to be an essential and sufficient inducer of extrinsic-mediated apoptosis *via* regulation of Fas-ligand expression in mature T cells [67]. Indeed, Lck-deficient T-cells were resistant to anticancer drugs and reconstitution of Lck activity recued sensitivity to drug-induced apoptosis [68]. Additionally, Lck-deficient cells exhibited pronounced resistance to apoptosis under ionizing radiation in a CD95-independent caspase-8 mediated mechanism [69]. Additionally, Lck was found to be constitutively activated in Nalm-6 cells [70]. Our results indicate that GB promotes the activation of Lck, and to a lesser extent Src, in Nalm-6 cells in a dose-dependent fashion. Thus, it is tempting to speculate that the apoptotic effect of GB could be mediated through activation of an Lck dependent pathway, which in turn promotes TNF-α production and eventual cell death *via* the extrinsic apoptotic pathway. However, additional studies are necessary to fully elucidate the complex mechanisms involved in GB mediated cytotoxicity, which may act in concert with additional effector molecules.

In conclusion, we demonstrate for the first time that GB possesses preferential anti-proliferative activity on a panel of malignant human leukemia/lymphoma cell lines compared with non-cancer cells. GB treatment disrupted cell-cycle progression in the BJAB cell line, which was manifested by inducing apoptotic DNA fragmentation and by G2/M phase arrest. GB treatment also resulted in PS translocation to the outer leaflet of the plasma membrane on B-lineage tumor cells (Nalm-6 and BJAB) and inflicted cytotoxicity, implicating the TNF-α → caspase-8 activation → caspase-3 activation → PARP-1 cleavage → DNA fragmentation signaling pathway that encompasses early-to-late biochemical events that are hallmarks of apoptosis. Importantly, caspase-8 and caspase-3 activation, as well as PARP-1 cleavage could be reversed by caspase inhibitors, providing additional evidence that GB is using an apoptotic pathway to induce cell death. Additionally, GB influenced Lck and Src activation that potentially could be participants in the pro-apoptotic mechanism in Nalm-6 cells. Hence, GB shows selective anti-proliferative activity and clear apoptosis-mediated cytotoxicity on B-lineage leukemia/lymphoma cell lines. These findings provide rational insight for further evaluation of GB as an anti-leukemia/lymphoma agent in animal models. Additional studies are required to elucidate the consequence of GB in combination with standard anti-cancer therapeutics.

## Supporting Information

Table S1 **Typical volumes of GB used in this study and their corresponding values in dry weight.**
(DOCX)
