# Supplementary material for: Searching in Mother Nature for Anti-Cancer Activity: Anti-Proliferative and Pro-Apoptotic Effect Elicited by Green Barley on Leukemia/Lymphoma Cells
Source: PLoS One. 2013 Sep 9;8(9):e73508. doi: 10.1371/journal.pone.0073508 (PMC3767772; doi:10.1371/journal.pone.0073508)
Supplement: Table S1 — Typical volumes of GB used in this study and their corresponding values in dry weight. (DOCX) [file pone.0073508.s001.docx]

**Supporting Information**

**Table S1. Typical volumes of GB used in this study and their**

**corresponding values in dry weight.**

| **Microliters (µl)**  **of GB** | **Corresponding values after lyophilization**  **in milligrams (mean ± standard deviation)** |
| --- | --- |
| 10 | 0.3 ± 0.009* |
| 50 | 1.5 ± 0.048 |
| 100 | 3.0 ± 0.009 |

*Values in milligrams were obtained after lyophilization from triplicates

as described in Materials and Methods
